# Intravascular versus surface cooling for targeted temperature management after out-of-hospital cardiac arrest – an analysis of the TTM trial data

**DOI:** 10.1186/s13054-016-1552-6

**Published:** 2016-11-26

**Authors:** Guy W. Glover, Richard M. Thomas, George Vamvakas, Nawaf Al-Subaie, Jules Cranshaw, Andrew Walden, Matthew P. Wise, Marlies Ostermann, Emma Thomas-Jones, Tobias Cronberg, David Erlinge, Yvan Gasche, Christian Hassager, Janneke Horn, Jesper Kjaergaard, Michael Kuiper, Tommaso Pellis, Pascal Stammet, Michael Wanscher, Jørn Wetterslev, Hans Friberg, Niklas Nielsen

**Affiliations:** 1 Department Intensive Care, Guy’s and St Thomas’ Hospital, King’s College London, London, UK; 2 Department of Intensive Care, University College Hospital, London, UK; 3 Department of Biostatistics, Institute of Psychiatry, Psychology and Neuroscience, King’s College London, London, UK; 4 Department of Intensive Care, St George’s Hospital, London, UK; 5 Department of Intensive Care, Royal Bournemouth Hospital, Bournemouth, UK; 6 Department of Intensive Care, Royal Berkshire Hospital, Reading, UK; 7 Adult Critical Care, University Hospital of Wales, Cardiff, UK; 8 Centre for Trials Research, College of Biomedical and Life Sciences, Cardiff University, Cardiff, UK; 9 Department of Neurology, Skåne University Hospital, Lund University, Lund, Sweden; 10 Department of Cardiology, Skåne University Hospital, Lund University, Lund, Sweden; 11 Department of Intensive Care, Geneva University Hospital, Geneva, Switzerland; 12 The Heart Center, Copenhagen University Hospital, Righospitalet, Copenhagen, Denmark; 13 Department of Intensive Care, Academic Medical Centre, Amsterdam, The Netherlands; 14 Department of Intensive Care, Medical Center Leeuwarden, Leeuwarden, The Netherlands; 15 Department of Intensive Care, Santa Maria degli Ángeli, Pordenone, Italy; 16 Department of Anesthesiology and Intensive Care, Centre Hospitalier de Luxembourg, Luxembourg City, Luxembourg; 17 Copenhagen Trial Unit, Center for Clinical Intervention Research, Copenhagen University Hospital, Rigshospitalet, Copenhagen, Denmark; 18 Department of Anesthesiology and Intensive Care, Skåne University Hospital, Lund University, Lund, Sweden; 19 Department of Anesthesiology and Intensive Care, Helsingborg Hospital, Helsingborg, Sweden; 20 Department of Critical Care, Guy’s and St Thomas’ NHS Foundation Trust, Kings Health Partners, Westminster Bridge Road, London, SE1 7EH UK

**Keywords:** Temperature, Hypothermia, Induced, Out-of-hospital cardiac arrest, Fever, Critical care, Shivering, Brain injuries

## Abstract

**Background:**

Targeted temperature management is recommended after out-of-hospital cardiac arrest and may be achieved using a variety of cooling devices. This study was conducted to explore the performance and outcomes for intravascular versus surface devices for targeted temperature management after out-of-hospital cardiac arrest.

**Method:**

A retrospective analysis of data from the Targeted Temperature Management trial. *N* = 934. A total of 240 patients (26%) managed with intravascular versus 694 (74%) with surface devices. Devices were assessed for speed and precision during the induction, maintenance and rewarming phases in addition to adverse events. All-cause mortality, as well as a composite of poor neurological function or death, as evaluated by the Cerebral Performance Category and modified Rankin scale were analysed.

**Results:**

For patients managed at 33 °C there was no difference between intravascular and surface groups in the median time taken to achieve target temperature (210 [interquartile range (IQR) 180] minutes vs. 240 [IQR 180] minutes, *p* = 0.58), maximum rate of cooling (1.0 [0.7] vs. 1.0 [0.9] °C/hr, *p* = 0.44), the number of patients who reached target temperature (within 4 hours (65% vs. 60%, *p* = 0.30); or ever (100% vs. 97%, *p* = 0.47), or episodes of overcooling (8% vs. 34%, *p* = 0.15). In the maintenance phase, cumulative temperature deviation (median 3.2 [IQR 5.0] °C hr vs. 9.3 [IQR 8.0] °C hr, *p* = <0.001), number of patients ever out of range (57.0% vs. 91.5%, *p* = 0.006) and median time out of range (1 [IQR 4.0] hours vs. 8.0 [IQR 9.0] hours, *p* = <0.001) were all significantly greater in the surface group although there was no difference in the occurrence of pyrexia. Adverse events were not different between intravascular and surface groups. There was no statistically significant difference in mortality (intravascular 46.3% vs. surface 50.0%; *p* = 0.32), Cerebral Performance Category scale 3–5 (49.0% vs. 54.3%; *p* = 0.18) or modified Rankin scale 4–6 (49.0% vs. 53.0%; *p* = 0.48).

**Conclusions:**

Intravascular and surface cooling was equally effective during induction of mild hypothermia. However, surface cooling was associated with less precision during the maintenance phase. There was no difference in adverse events, mortality or poor neurological outcomes between patients treated with intravascular and surface cooling devices.

**Trial registration:**

TTM trial ClinicalTrials.gov number https://clinicaltrials.gov/ct2/show/NCT01020916NCT01020916; 25 November 2009

## Background

Targeted temperature management (TTM) may be indicated in patients who remain comatose following return of spontaneous circulation (ROSC) after out-of-hospital cardiac arrest (OHCA) [1, 2].

TTM may be delivered by conventional or active methods. Conventional methods include exposure, cold intravenous fluids or ice slurry/proprietary cooling pads [3–5]. Active methods are microprocessor-controlled devices where the thermal energy is regulated by the patient’s temperature, via a closed-feedback loop, delivering a programmed rate of temperature change and/or target temperature. Active devices may be invasive/intravascular (IV) catheters [6–8] or non-invasive/surface devices (SFC), such as water-circulating blankets [9, 10] or hydrogel pads [11, 12]. Cooling methods have been reviewed comprehensively [4, 13–15].

TTM is divided into induction, maintenance, rewarming and fever control phases. Ideal devices achieve target temperature quickly, allow for accurate maintenance and slow, controlled rewarming. Conventional methods are associated with overcooling and rebound hyperthermia [16–18]. Active devices deliver more rapid induction, with less temperature variation compared to conventional [19–22], however, there may be significant differences in performance and adverse effects between SFC and IV [11, 19, 23–27]. IV and SFC cooling may be mechanistically different. IV cooling of circulating blood could be likened to convective cooling. Whilst SFC also relies on perfusion to transfer cold from the periphery to the core, this will be affected by vasoconstriction and there may be a significant element of conductive cooling. Aside from any differences in controlling core body temperature, there may be other effects. The direct contact between the cold catheter and blood is non-physiological; this could have effects on blood rheology [28], which could be harmful in the context of myocardial and cerebral ischaemia; at the same time, direct circulation of cold blood into the right heart may have a protective effect on the myocardium [29]. In comparison SFC cooling is more ‘physiological’. The human body is conditioned to receive changes in thermal input from the environment primarily through the skin, and any homeostatic mechanisms are designed to respond to this type of thermal challenge.

The aim of this study was to investigate IV versus SFC devices for TTM after OHCA. Our hypothesis was that there is no difference in TTM performance, adverse events or patient-centred outcomes.

## Methods

A post hoc analysis of data from the Targeted Temperature Management at 33 °C versus 36 °C after Cardiac Arrest trial (TTM trial; NCT01020916) [30]. This randomized controlled trial of two levels of temperature management after OHCA recruited 950 patients in 36 centres in Europe and Australia between November 2010 and January 2013. The TTM trial protocol was approved by ethics committees in each participating country, and informed consent was waived or was obtained according to national legislation, in line with the Helsinki declaration. The trial demonstrated no difference in all-cause mortality at the end of the TTM trial.

Adult patients resuscitated from OHCA of a presumed cardiac cause, who remained unconscious, were included in the TTM trial. All patients were sedated and received invasive mechanical ventilation. Core temperature was measured primarily via a urinary catheter. Temperature was managed with either an IV or SFC system according to centre preference. Centres could use one or both types of device but the type had to be decided and recorded in the case report form prior to randomization. The intervention was divided into three periods (a) achievement of target temperature (4 hours); (b) maintenance of target temperature (24 hours); and (c) rewarming to 37 °C (8 hours). Following randomization, immediate measures were taken to achieve the target temperature. For patients allocated to 36 °C whose initial temperature was below 36 °C, the temperature was allowed to passively rise before it was maintained at target. Twenty-eight hours after the start of the intervention, the device was set to raise the temperature to 37 °C, with a maximum speed of 0.5 °C/hour. After 36 hours, sedation was discontinued or tapered and the patients were allowed to recover. Other aspects of patient management were according to standard practice.

Exclusions from this study were (i) patients withdrawn without receiving an intervention (ii) patients with device type not recorded (iii) patients with no initial temperature recorded (iv) patients whose initial temperature was below, or within 1 °C of the allocated target (excluded from induction analysis) (v) patients who were rewarmed early (excluded from maintenance analysis) (vi) patients who died during the intervention (excluded from analysis of performance and adverse events but not from mortality).

Performance was analysed as follows:

**Induction** (33 °C group)

i. Maximum cooling rate
ii. Time from randomization to target temperature. The temperature of 33.5 °C was chosen, halfway between the trial target and the upper limit recommended by the International Liaison Committee on Resuscitation (I LCOR) of 34 °C (2010 recommendations) [31]
iii. Number of patients achieving 33.5 °C
iv. Episodes of overcooling (temperature ≤32.5 °C during the first 8 hours; a pragmatic value between the trial target and the lower limit recommended by ILCOR)

**Maintenance and rewarming phase** (33 °C and 36 °C groups)

i. Cumulative deviation from target temperature (magnitude × duration of deviation)
ii. Number of patients with temperature reading +/−0.5 °﻿C ﻿﻿out of range
iii. Time out of range
iv. Number of patients with temperature ≥37.5 °C

Adverse events were defined according to the TTM trial protocol [32]. Patient-centred outcomes from the TTM trial were also assessed; all-cause mortality through to the end of the trial, and the composite of poor neurological function or death at 180 days, as evaluated with the Cerebral Performance Category (CPC) scale 3–5 and the modified Rankin scale (mRs) 4–6.

Additionally, the TTM trial sites were surveyed to record which cooling device they used and other aspects of their cooling practice, including their use of additional cooling methods. The survey was administered using commercially available subscription software (SurveyMonkey®, Palo Alta, CA, USA).

### Statistical analysis

Analyses were undertaken in STATA v.14 (Stata Statistical Software, College Station, TX, USA). For baseline characteristics, categorical variables are presented as counts and percentage and were analysed using the chi-square test, normally distributed data are expressed as mean ± standard deviation (SD) and were analysed using the Student’s *t* test and skewed data are expressed as median and interquartile range [IQR] and were analysed using the Wilcoxon's rank-sum test.

Performance and outcome data were analysed as clustered (*xtset*) at the level of the TTM centres, within which data existed. Continuous outcomes were analysed using mixed effects regression (*xtreg*). Binary outcomes were analysed using mixed effects logistic regression (*xtlogit*). The number achieving temperature ever and number out of range were analysed as over-dispersed count data using mixed effects negative binomial regression (*xtnbreg*). Transformations were used to normalize the continuous outcomes.

The analyses obtained estimates of the group differences, under the assumption of data missing completely at random. No adjustment for potential confounders was made for the treatment effects. All treatment effects were produced using a fixed effects estimator (*fe*). Continuous outcomes are reported as Cohen’s d effect size estimates, which used the pooled standard deviation. Their confidence intervals were obtained through the *lincom* post-estimation command. Effect sizes for the binary and count data are reported as log-odds ratios and log-risks ratios. All tests were two-tailed and a *p* value of <0.05 was considered significant.

## Results

Twenty-eight (77%) centres used SFC devices and ten (28%) used IV. All IV centres used the Thermoguard (Thermoguard, ZOLL Medical Corporation, Chelmsford, MA, USA). There was variation among SFC centres and some used more than one type: Arctic Sun, ten (Arctic Sun, Medivance, Louisville, CO, USA); Blanketrol, five (Blanketrol, Cincinnati Sub-Zero, Cincinnati, OH, USA); Allon/Criticool, 11 (MTRE Advanced Technologies Ltd, Rehovot, Israel) and other, four.

The cooling device was initiated in the intensive care unit (ICU) in 31 centres with four starting in the emergency department and one in the cardiac catheter laboratory. Additional conventional cooling (e.g. cold fluids, ice packs) was used for induction with varying frequency (always 26%; frequently 26%; occasionally 20%; rarely 11%; never 17%). Additional rescue cooling was not commonly required (always 0%; frequently 0%; occasionally 22%; rarely 36%; never 42%).

Of the 950 patients in the TTM trial, 934 were available for analysis having excluded those who were withdrawn (three in the 33 °C group, eight in the 36 °C group) or did not have the device recorded (five in the 36 °C group). There were 240 (26%) IV versus 694 (74%) SFC.

Baseline characteristics of the population are shown in Table 1.

**Table 1:** Baseline characteristics of the intravascular and surface device patients

Characteristic	Intravascular *n* = 240	Surface device *n* = 694	*p* value
Age (years)	63 **±** 12.7	64.5 **±** 12.0	0.12
Male gender	190, 79%	567, 82%	0.39
Body mass index (kg/m^2^)	26.9 **±** 5.0	27.0 **±** 8.1	0.78
Bystander witnessed	212, 89%	623, 90%	0.64
Bystander CPR	162, 68%	518, 74%	0.04
Shockable rhythm	187, 78%	547, 79%	0.78
Time to CPR (minutes)	1 [0–2]	1 [0–2]	0.37
Time to ROSC (minutes)	25 [17-39]	25 [17-40]	0.73
Time collapse to randomization (minutes)	176 [125–227]	158 [114–210]	0.92
Baseline temperature on admission (°C)	35.2 **±** 1.2	35.3 **±** 1.2	0.19
GCS	4 [3-5]	3 [3-5]	0.31
Serum pH	7.20 **±** 0.15	7.20 **±** 0.16	0.65
Serum lactate (mmol/l)	6.3 **±** 4.3	6.9 **±** 4.5	0.07
Circulatory shock on admission	42, 18%	94, 14%	0.13
Coronary angiography	181, 75%	404, 58%	0.27
Haemodialysis on day 1	9, 4%	21, 3%	0.50
SOFA - C	3 [2-4]	3 [2-4]	0.26

Values are mean ± SD, n,% or median [IQR] as appropriate

*Abbreviations*: *CPR* cardiopulmonary resuscitation, *ROSC* return of spontaneous circulation, *GCS* Glasgow Coma Scale, *SOFA - C* Cardiovascular Sequential Organ Failure Assessment

**Fig. 1:**
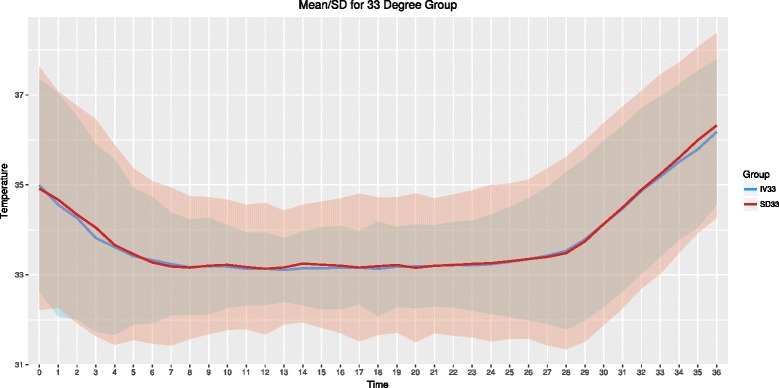
Patient temperature for the 33 °C group over the intervention periods. Mean and standard deviation temperature (°C). *Blue line and shading* is intravascular group, *red line and shading* is surface group. Time in hours

**Fig. 2:**
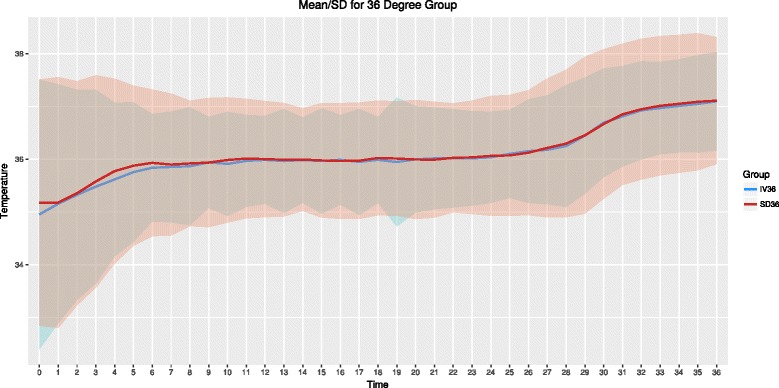
Patient temperature for the 36 °C group over the intervention periods. Mean and standard deviation temperature (°C). *Blue line and shading* is intravascular group, *red line and shading* is surface group. Time in hours

The mean ± SD temperature for IV and SFC devices during the intervention are presented graphically (Figs. 1 and 2).

**Fig. 3:**
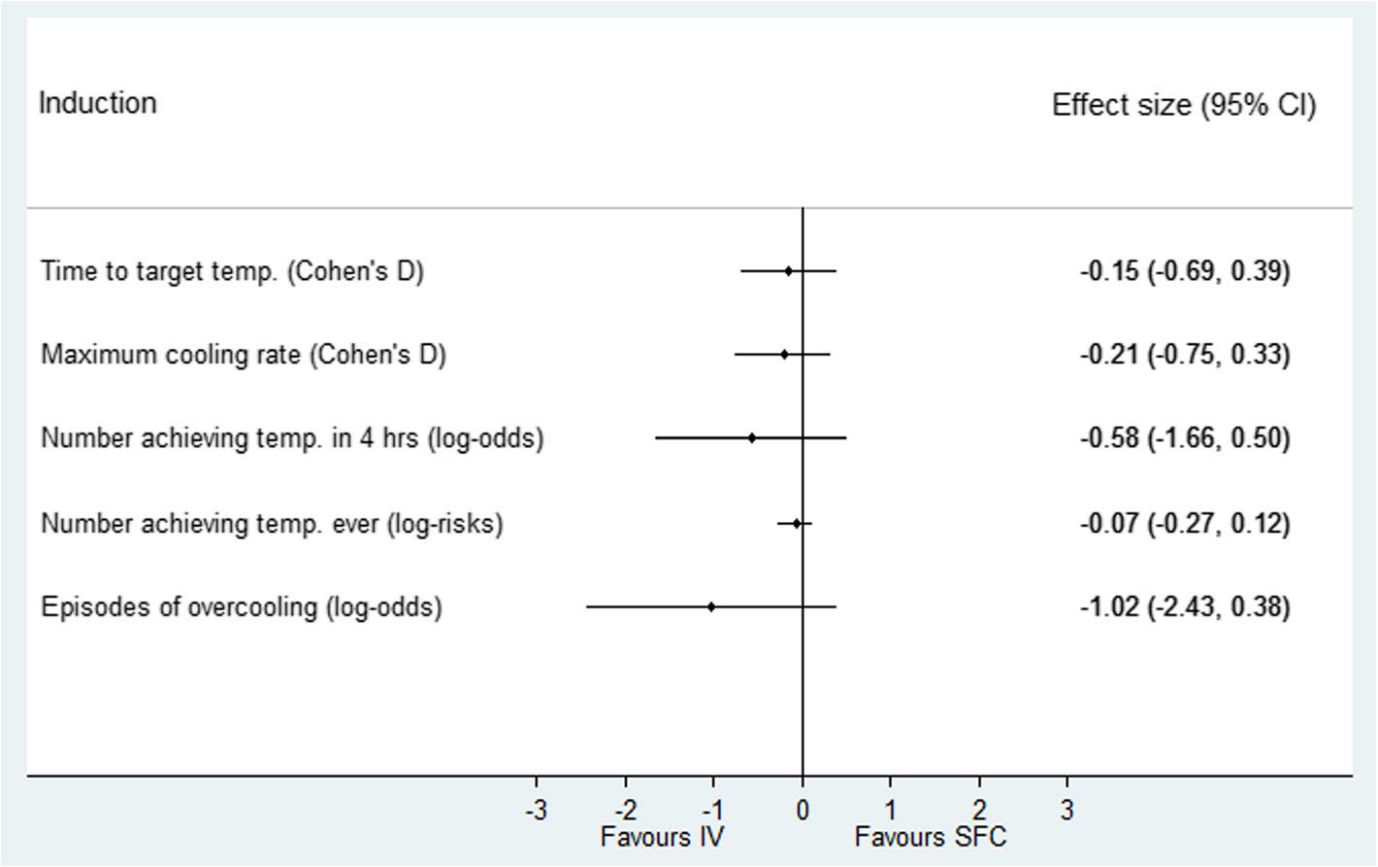
Performance of surface versus intravascular devices in induction phase. Effect estimates and 95% confidence intervals. *SFC* surface device, *IV* intravascular

For the induction analysis, 195 patients were available after exclusions. Results are shown in Table 2. Effect estimates (Fig. 3) show that patients treated either with SFC or IV took the same time to reach target temperature (*p* = 0.58). No treatment difference was detected for maximum cooling rate *(p* = 0.44). There was no difference between the cooling devices in terms of the number of patients achieving the target temperature in 4 hours or ever during the intervention (*p* = 0.30 and *p* = 0.47). Finally, although there were more episodes of overcooling with SFC, the difference was not significant (*p* = 0.15).

**Table 2:** Efficacy of the intravascular versus surface devices during induction and maintenance/rewarming phases

Performance metric		Intravascular	Surface device	*p* value
Induction (33 °C group; *n* = 195); 52 intravascular versus 143 surface)				
Time to target temperature (minutes)		210 [180]	240 [180]	0.58
Maximum cooling rate in induction phase (°C/hour)		1 [0.7]	1 [0.9]	0.44
Number of patients achieving target temperature in 4 hours		34, 65%	86, 60%	0.30
Number of patients achieving target temperature ever		52, [100]	140, [97]	0.47
Episodes of overcooling		4, 8%	49, 34%	0.15
Maintenance and rewarming (33 and 36 °C groups; n = 844); 226 intravascular versus 618 surface)				
Cumulative deviation out of range (°C hours)		3.2 [5.0]	9.3 [8.0]	<0.001
Number of patients out of range		127, 57.0%	568, 91.5%	0.006
Time out of range (hours)		1 [4.0]	8.0 [9.0]	<0.001
Number of patients with temperature ≥37.5 °C	33 °C	4, 3%	41, 12%	0.44
	36 °C	27, 23%	153, 47%	0.99

Values are n,% or median [IQR] as appropriate

**Fig. 4:**
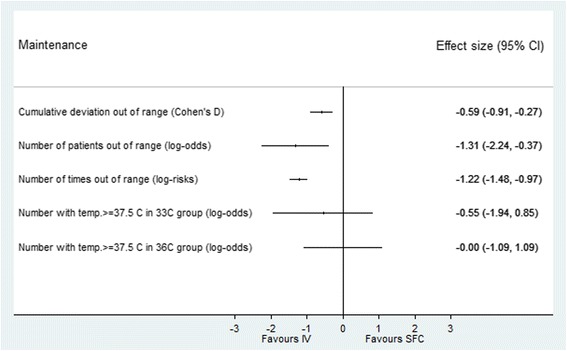
Performance of surface versus intravascular devices in maintenance phase. Effect estimates and 95% confidence intervals. *SFC* surface device, *IV* intravascular

In the maintenance phase, 844 patients were available for analysis. Results are shown in Table 2. Effect estimates (Fig. 4) show that the cumulative deviation from target temperature was significantly higher for the SFC group (Cohen’s d effect size 0.59 (CI −0.91, −0.27; *p* < 0.001)). More patients in the SFC group were out of range during maintenance (log-odds effect size −1.31 (CI −2.24, −0.37; *p* = 0.006)) as was the median time out of range (log-risks effect size −1.22 (CI −1.48, −0.97; *p* < 0.001)). A greater proportion of SFC patients had a temperature >37.5 °C but this was not statistically significant.

**Fig. 5:**
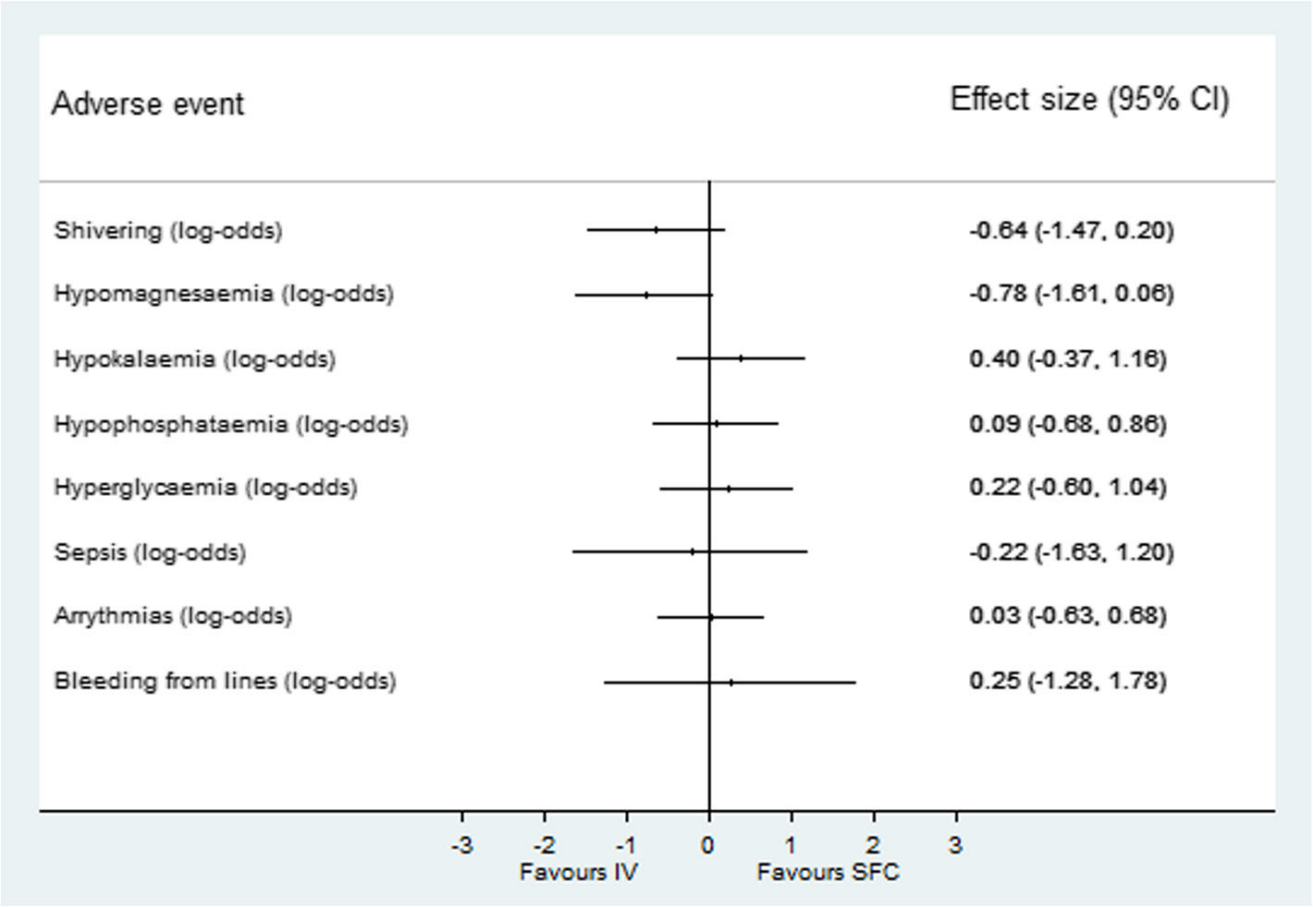
Adverse events for surface versus intravascular devices. Effect estimates and 95% confidence intervals. *SFC* surface device, *IV* intravascular

There was no statistically significant difference in any of the adverse outcomes between the SFC and IV groups (Fig. 5). The TTM trial primary outcome of all-cause mortality at the end of the trial was the same in both groups (46.3% in IV vs. 50.0% in SFC; *p* = 0.32). There was no difference in the secondary trial outcomes of CPC scale 3–5 (IV 49.0% vs. SFC 54.3%; *p* = 0.18) or mRs 4–6 (IV 49.0% vs. SFC 53.0%; *p* = 0.48).

## Discussion

In this study there was no difference between IV and SFC in the induction phase of TTM. A maximum cooling rate of 1.0 °C hr^−1^ was achieved in both groups. Whilst rates up to 3 °C hr^−1^ have been claimed for some devices [4, 14, 33], most studies have been in the region of 1–1.5 °C hr^−1^, with similar performance between IV and surface devices [10, 12, 19, 34, 35].

For patients managed at 33 °C, the median time to achieve target temperature was similar. This represents the time from randomization to target temperature and includes the time taken to initiate cooling as well as the time with cooling applied. Although all patients were recruited within 4 hours of ROSC, we were unable to determine the exact time that the cooling device was started and there may have been a staggered start, as many centres used additional cooling methods for induction. Cold intravenous fluids may significantly reduce the time to achieve target temperature [36, 37]. As such, the cooling rate that we have reported may either underestimate (cooling device started later), or overestimate (significant cooling effect from additional methods) the actual device performance. Whilst we cannot exclude an imbalance in the use of other cooling methods between SFC and IV centres, our survey indicated that additional cooling methods were applied more commonly in the SFC centres than in IV (unpublished data). Whether this reflects random variation, or whether this is deliberate because of a perception of inferior performance by SFC centres is not known from this data. Any future studies of IV versus SFC devices should rigorously control for the use of additional cooling methods.

It is difficult to compare our data with the published literature as other studies have used different targets and have variably reported the start time as ROSC, hospital admission or time when cooling started. Nevertheless, median times of 190–450 minutes to achieve target are reported [9, 18, 21, 24, 27, 33, 34, 38–40].

Delays to initiate cooling of between 1 and 3 hours are common [24, 27, 34, 38–40] and this may be longer with IV than with conventional and/or surface devices [27, 34].

Overall, there was no difference in the proportion of patients reaching target temperature during the intervention. Although only 60–65% of patients achieved target temperature within 4 hours, the TTM trial still outperformed other OHCA trials. In the Hypothermia after Cardiac Arrest study only 25% of patients reached a temperature <34 °C within 4 hours and almost 20% never reached their target [39]. Time to reach target may decrease with experience [9, 41].

Overcooling during the induction phase was proportionally more common with SFC than with IV but the result was not statistically significant, possibly due to the reduced numbers available for analysis. A recent study has demonstrated a similar but significant result [25]. It is known that overcooling is common when using conventional cooling [17], and whilst this may be reduced using SFC devices [16], there are significant differences between different SFC technologies [11]. The clinical significance of these overcooling events is not known. Overcooling of up to 1 °C below target may be acceptable, provided that the temperature remains greater than 30 °C [4]. We chose to apply relatively strict criteria in order to explore subtle differences in device performance. Is there an explanation for possible inferior performance in SFC devices? One theory is that it could relate to the modality of surface cooling per se, because of a time lag in the equilibration of temperature from the peripheral compartment to the core where temperature is monitored. Alternatively, it may be a function of the device feedback algorithms.

In this study, the IV group had significantly less deviation from the target temperature during the maintenance phase than the SFC group. Whether we analysed the number of patients ever out of range, the time out of range or the cumulative temperature deviation, all of these results were highly significant. This is consistent with the published literature to date [19, 26]. The time out of range for SFC is notably high; whilst this is influenced by the relatively low tolerance of our definition (0.5 °C), the cause may include lack of power in SFC devices or the time lag in equilibration discussed above, especially under conditions of vasoconstriction.

Temperature deviation is generally less than 1 °C and the clinical significance of this is unclear. It has been recommended that in the maintenance phase, temperature should be tightly controlled, with minor or no fluctuations (maximum 0.2 °C to 0.5 °C) but this is not based on high-quality evidence [4]. In patients managed at 33 °C, modest deviations will still remain within the recommended range of 32–36 °C. However, in patients managed at 36 °C, deviation may allow patients to exceed the fever threshold. Even with the best technologies, there may be a small number of outliers with significant temperature deviation [38]. Despite a protocol designed to avoid fever in both arms, a quarter of patients had one or more readings >37.5 °C. This was more common in the 36 °C group as compared to 33 °C group.

In this study, there was no statistically significant difference in adverse events, although there was a trend towards more shivering and hypomagnesaemia in SFC patients. Previous studies that have shown more shivering with surface devices [23, 24], perhaps related to the role of cutaneous thermoreceptors in the shivering reflex. Our findings contradict other studies, which have demonstrated more electrolyte disturbance and sepsis with IV cooling [24, 25]. The reasons for this are not known, but could reflect the fact that experienced centres in the TTM trial may have managed patients with protocols for electrolyte management and prophylactic antibiotics.

Data on adverse effects from cooling devices were not specifically collected during the TTM trial. Reported adverse events include skin damage [42, 43], deep vein thrombosis [38, 44, 45] and bleeding [26, 34, 46]. The safety profile of devices may influence clinician’s choice and may influence performance if adverse events require discontinuation. Any future studies should prospectively collect data on the occurrence of these important outcomes.

Mortality and poor neurological outcomes were not significantly different between IV and SFC. This supports previous literature, which shows no difference in levels of biomarkers of neurological injury [26], or in outcome [20, 24, 26, 27]. Whilst Oh and colleagues observed that SFC was associated with poor neurological outcome and increased hospital mortality when compared to IV, this was no longer apparent after propensity score matching [25]. The recent ICEREA study demonstrated that whilst IV cooling was technically superior to basic external cooling, it did not lead to statistically significant reductions in mortality [27]. Patient-centred outcomes related to TTM devices may not be limited to the effect of body temperature control or even to the incidence of clinical side effects. As we have discussed, there may be complex and compounding effects due to haemodynamic consequences of vasoconstriction and shivering on the injured brain or myocardium, unmeasured rheological effects, or consequences of more rapid myocardial cooling. These remain areas for further study.

The results of the TTM trial, and the recent change in the ILCOR recommendation to a target temperature of 32–36 °C may, on the face of it, make any small differences in device performance during maintenance appear less relevant. On the other hand, if more clinicians are targeting 36 °C, accurate temperature control to avoid the risk of pyrexia may be of greater importance.

This study is the largest to date, investigating the difference between SFC and IV devices. A wide range of device-related metrics were analysed, and performance was assessed at two different temperatures. Whilst this was an observational study, the data was prospectively collected as part of a high-quality randomized controlled trial. Because all centres were experienced in TTM, and selected the device according to their own preference, it is likely that they had significant experience of the device and as such use should have been optimal. This study is investigator led and received no commercial or industry funding.

This study has limitations. As with all observational studies, there is a risk of confounding due to unmeasured differences and other factors which influence patient temperature may have varied (e.g. sedation or neuromuscular blockade, selection of catheters, pads or blankets, troubleshooting device problems or additional cooling methods).

Whilst the TTM trial was very well conducted, the data set was not fully complete and a number of patients were excluded due to missing data. The authors acknowledge that the random effects estimator is more efficient, however, lack of adequate data on both the individual and cluster levels led to the choice of the fixed effects estimator, which is more robust against selection bias.

Finally, we have analysed cooling devices by category rather than comparing proprietary devices because we did not have patient-level data on specifically which device was used. It is possible that there is heterogeneity between different SFC devices and this study cannot exclude differences in performance between the SFC devices used. The study was not designed to compare intravascular or surface devices with basic external cooling and whilst there is evidence that this is logistically and technically inferior, it does remain in use in some centres.

## Conclusions

In patients who remain comatose following successful resuscitation after OHCA, TTM may be applied with either intravascular or surface cooling devices. Intravascular and surface cooling was equally effective during the induction of mild hypothermia. However, surface cooling was associated with less precision during the maintenance phase. Although the clinical significance of this is not known, this may have implications for the management of patients at 36 °C. There was no statistically significant difference in adverse events, mortality or poor neurological function between patients treated with intravascular and surface cooling devices.

## Abbreviations

CPC: Cerebral Performance Category
GCS: Glasgow Coma Scale
IV: intravascular device
mRs: modifed Rankin scale
OHCA: out-of-hospital cardiac arrest
ROSC: return of spontaneous circulation
SFC: surface device
SOFA - C: Cardiovascular Sequential Organ Failure Assessment
TTM: targeted temperature management
